# Stat3 is a positive regulator of gap junctional intercellular communication in cultured, human lung carcinoma cells

**DOI:** 10.1186/1471-2407-12-605

**Published:** 2012-12-18

**Authors:** Mulu Geletu, Rozanne Arulanandam, Samantha Greer, Aaron Trotman-Grant, Evangelia Tomai, Leda Raptis

**Affiliations:** 1 Departments of Microbiology and Immunology and Pathology, Queen’s University, Kingston, Ontario, K7L3N6, Canada; 2 Present address: Center for Innovative Cancer Research, Ottawa Hospital Research Institute, 501 Smyth Road, Ottawa, ON K1H 8L6, Canada; 3 Present address: Department of Laboratory Medicine and Pathobiology, University of Toronto, Toronto, Ontario, Canada, M5S1A8; 4 Present address: Institute for Physiology and Pathophysiology, Medical Faculty, University of Heidelberg, Im Neuenheimer Feld 326, Heidelberg, D-69120, Germany

**Keywords:** Stat3, Electroporation, Indium-Tin oxide, Gap junctions, Src, Cell to cell adhesion, Lung cancer

## Abstract

**Background:**

Neoplastic transformation of cultured cells by a number of oncogenes such as *src* suppresses gap junctional, intercellular communication (GJIC); however, the role of Src and its effector Signal transducer and activator of transcription-3 (Stat3) upon GJIC in non small cell lung cancer (NSCLC) has not been defined. Immunohistochemical analysis revealed high Src activity in NSCLC biopsy samples compared to normal tissues. Here we explored the potential effect of Src and Stat3 upon GJIC, by assessing the levels of tyr418-phosphorylated Src and tyr705-phosphorylated Stat3, respectively, in a panel of NSCLC cell lines.

**Methods:**

Gap junctional communication was examined by electroporating the fluorescent dye Lucifer yellow into cells grown on a transparent electrode, followed by observation of the migration of the dye to the adjacent, non-electroporated cells under fluorescence illumination.

**Results:**

An inverse relationship between Src activity levels and GJIC was noted; in five lines with high Src activity GJIC was absent, while two lines with extensive GJIC (QU-DB and SK-LuCi6) had low Src levels, similar to a non-transformed, immortalised lung epithelial cell line. Interestingly, examination of the mechanism indicated that Stat3 inhibition in any of the NSCLC lines expressing high endogenous Src activity levels, or in cells where Src was exogenously transduced, did not restore GJIC. On the contrary, Stat3 downregulation in immortalised lung epithelial cells or in the NSCLC lines displaying extensive GJIC actually suppressed junctional permeability.

**Conclusions:**

Our findings demonstrate that although Stat3 is generally growth promoting and in an activated form it can act as an oncogene, it is actually ***required*** for gap junctional communication both in nontransformed lung epithelial cells and in certain lung cancer lines that retain extensive GJIC.

## Background

Gap junctions are plasma membrane channels that provide a path of direct communication between the interiors of neighboring cells and are formed by the connexin (Cx) family of proteins. An increase in cell proliferation correlates with a reduction in gap junctional, intercellular communication (GJIC [1]). In fact, a number of oncogene products such as v-Src [2], the polyoma virus middle Tumor antigen, an oncogene which acts by activating Src family kinases (mT [3,4]), the chaperone Hsp90N [5], vRas [6,7] and others have been shown to interrupt junctional communication.

Extensive evidence has indicated that expression of the Src tyrosine kinase leads to gap junction closure, through phosphorylation of the ubiquitous connexin, Cx43. Src exerts its effect either through direct tyrosine phosphorylation of Cx43, or indirectly, through activation of the serine/threonine, Erk1/2 or protein kinase C family kinases [8]. Examination of levels of tyr-418 phosphorylated, ie activated Src in a number of Non Small Cell Lung Cancer (NSCLC) biopsies previously showed the presence of higher Src activity than the surrounding, non-tumor lung tissue [9,10]. However, Src’s contribution to GJIC suppression in NSCLC lines and primary cells which may express other oncogenes in addition to Src, or different levels of Src effectors, remains to be determined.

The Signal Transducer and Activator of transcription-3 (Stat3), an important Src downstream effector, is a cytoplasmic transcription factor. Following phosphorylation on tyr-705 by Src, as well as by growth factor or cytokine receptors such as the IL6 family, Stat3 normally dimerises through a reciprocal SH2 domain-phosphotyrosine interaction and translocates to the nucleus, where it induces the transcription of specific genes [11]. The effect of Src upon Stat3 activation in NSCLC lines is at present unclear. Examination of Stat3 levels in certain NSCLC lines demonstrated that Src is a major Stat3 activator, transducing signals from EGFR and IL6 that lead to apoptosis inhibition [12], while in another report [13] Src inhibition in different NSCLC lines was found to actually ***increase*** Stat3-ptyr705. However, we and others previously demonstrated that cell-to-cell adhesion, as observed at confluence of cultured cells, causes a dramatic increase in Stat3 activity levels in a number of cellular systems ( [14-16] reviewed in [17]); for this reason, cell density must be taken into account in the examination of the effect of different factors such as Src upon Stat3 activity levels. In the present report this was achieved by measuring Stat3-ptyr705 phosphorylation and activity levels at a range of densities.

We previously assessed GJIC in a number of lung cancer lines [18]. In the present work GJIC was examined using an apparatus where cells were grown on a glass slide, half of which was coated with electrically conductive, optically transparent, indium-tin oxide. An electrode was placed on top of the cells and an electrical pulse, which opens transient pores on the plasma membrane, was applied in the presence of the fluorescent dye, Lucifer yellow. Although this technique is adequate for a number of lines, the turbulence generated as the electrode is removed can cause cell detachment, which makes GJIC examination problematic. Here, we revisited the question of GJIC levels in lung cancer lines using an improved technique, where the upper electrode is eliminated. This approach is valuable for the electroporation of tumor-derived lines especially at high densities, where cell adhesion to the substratum may be weak. Interestingly, the results revealed that cell density *per se* triggers a dramatic increase in both Cx43 levels and GJIC. Two NSCLC lines, QU-DB and SK-LuCi6 were found to have extensive GJIC, similar to control, nontransformed lung epithelial cells, while GJIC in five other lines was very low or undetectable. Investigation of the mechanism of gap junction closure revealed an inverse relation between Src activity levels and GJIC. Further studies led to the discovery that, unlike Ras inhibition in Src-transformed fibroblasts [19], Stat3 inhibition in NSCLC lines with high Src activity does not restore GJIC. On the contrary, Stat3 inhibition in lines displaying extensive GJIC (QU-DB, SK-LuCi6) suppressed junctional permeability, indicating that Stat3 activity is actually ***required*** for the maintenance of gap junction function in these lung cancer lines.

## Results

### Cell density upregulates GJIC and connexin-43 protein levels

**Figure 1:**
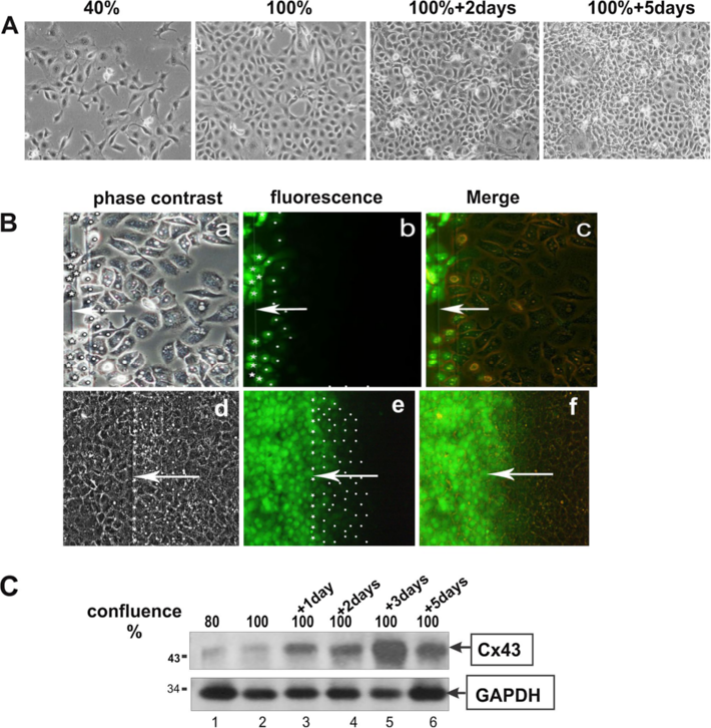
**Cell density increases GJIC and Cx43 levels.A**. Immortalised lung epithelial E10 cells were plated in 3 cm plastic petri dishes, grown to different densities and photographed under phase-contrast illumination. Magnification: 240x. **B**. E10 cells were plated in electroporation chambers and subjected to a pulse in the presence of Lucifer yellow when 90% confluent (***a-c***) or 3 days after confluence (***d-f***) and photographed under phase-contrast (***a, d***), fluorescence (***b, e***) or combined (***c, f***) illumination (see Methods, Figure 7). Arrows point to the position of the edge of the electroporated area. In ***a***, ***b***, ***d*** and ***e***, stars mark cells loaded with the dye at the edge of the electroporated area and dots mark cells into which the dye was transferred through gap junctions. Magnification: 240x **C**. E10 cells were seeded in plastic petri dishes and when they reached the indicated densities, detergent cell extracts were probed for Cx43 (top) or GAPDH (bottom) as a control.

A number of reports showed that gap junction function is dependent upon cell to cell contact and the assembly of adherens junctions [20,21]. Since the opportunity for engagement of cadherins, key components of adherens junctions, is expected to increase with cell density, we examined the effect of cell density upon GJIC. To this effect, we took advantage of the nontransformed mouse lung epithelial type II line, E10 that has extensive GJIC, an even and flat morphology and good adhesion to the substratum even at high densities [22] (Figure 1A). In addition, unlike nontransformed human lung lines such as NL-20 [23], E10 cells can be grown in the absence of growth factors that could affect GJIC. Cells were plated in electroporation chambers and when 90% confluent or at 3 days post-confluence Lucifer yellow was electroporated and the movement of the dye through gap junctions observed under fluorescence and phase contrast illumination (see Methods). The results are presented as the average number of cells where dye transfered, per cell loaded with the dye by electroporation (GJIC). As shown in Figure 1B, ***a-c***, although cells at 90% confluence did display some gap junction transfer (GJIC ~1.5), GJIC increased to ~6 at 3 days post-confluence (Figure 1B, ***d-f***), indicating that cell density causes a dramatic increase in GJIC (Table 1,A).

**Table 1:** Effect of Stat3 downregulation upon GJIC

**A. Cells with extensive junctional communication**						
**Cell line**	**Treatment**^**α**^	**Src**^**β**^**(%)**	**Stat3**^**β **^**(%)**		**GJIC**^**γ**^	
			**50%**	**100+3d**	**90%**	**100+3d**
**E10**	**-**	6±1	9±3	26±9	1.5±0.5	6.0±1
**“**	**DMSO**	6±1	9±3	30±8	-	6.0±1
**“**	**CPA7**	5±1	2±1.1	3±1	-	0.2±0.1
**“**	**sh-Stat3**	N/A	6±1.1	8±2	-	1.0±0.2
**QU-DB**	**-**	7±1	10±2	20±4	1±0.2	6.3±1
**“**	**DMSO**	7±1	10±2	22±3	-	6.3±1
**“**	**CPA7**	5±1	2±0.5	2±0.5	-	0.2±0.1
**“**	**sh-Stat3**	N/A	5±3	8±2	-	0.8±0.2
**SK-LuCi6**	**-**	5±1	8±2	21±4	1.8±0.2	6.5±1
**“**	**DMSO**	5±1	8±2	20±5	-	6.5±1
**“**	**CPA7**	5±1.2	2.8±1.25	3±1	-	0.3±0.1
**“**	**sh-Stat3**	N/A	6±2	4±0.5	-	1±0.2
**“**	**Jak inhib.1**	5.2±0.3	4.2±1.1	5±0.5	-	0.5±0.2
**“**	**Stat3C**	5.1±1	22±9	97±10	-	8±1
**B. Cells expressing activated Src**						
**A549**	**-**	95±11	93±12	320±32	0.1 ±0.1	0.3 ±0.1
**“**	**DMSO**	95±11	93±12	320±32	-	0.3 ±0.1
**“**	**CPA7**	93±10	8±1	12±2	-	0.1 ±0.1
**“**	**sh-Stat3**	N/A	12±3	11±4	-	0.1±0.1
**E10-*Src***	**DMSO**	98±12	98±15	350±28	-	0.4 ±0.2
**“**	**CPA7**	95±11	5±1	15±4	-	0.1 ±0.1
**“**	**sh-Stat3**	N/A	9±3	20±3	-	0.1±0.1
**SK-LuCi6-*Src***	**DMSO**	100±12	100±12	420±33	-	0.2±0.1
**“**	**CPA7**	98±10	3±1	9±1	-	0.1 ±0.1
**“**	**sh-Stat3**	N/A	11±3	17±3	-	0.3±0.1
**“**	**sh-Stat3+Das.**	4±1	6±1.1	14±3	-	0.2±0.1
**SK-Lu-1**	**DMSO**	85±5	90±11	311±23	-	1±0.2
**“**	**CPA7**	82±4	6±1	8±3	-	0.1 ±0.1
**CALU-1**	**DMSO**	96±9	100±10	290±12	-	0.1 ±0.1
**“**	**CPA7**	90±12	8±2	6±1	-	0.1 ±0.1
**SW-900**	**DMSO**	100±13	100±12	405±21	-	0.1 ±0.1
**“**	**CPA7**	96±11	12±1	11±2	-	0.1 ±0.1
**CALU-6**	**DMSO**	95±11	93±10	300±18	-	0.1 ±0.1
**“**	**CPA7**	93±10	10±1	15±5	-	0.1 ±0.1

^**α**^For Stat3 inhibition, cells were treated with 50 μM CPA7, or the DMSO carrier for 24 hrs, or infected with a lentivirus vector expressing a Stat3-specific, shRNA [37]. For Stat3 upregulation, cells were infected with a retroviral vector containing Stat3C. Jak inhibitor-1 was used at 5μM [16].

^**β**^ Stat3-tyr705 or Src-ptyr418 levels were measured by Western blotting. Numbers represent relative values obtained by quantitation analysis. Averages of at least three experiments ±SEM are shown. For Stat3, data from cells grown to 50% confluence or 3 days after confluence are presented [15], with the average of the values for DMSO-treated, Src-transduced, SK-LuCi6-***Src*** cells grown to 50% confluence taken as 100%. The transcriptional activity values obtained paralleled the Stat3-705 phosphorylation levels indicated (Figure 4C and D, see Methods).

^**γ**^GJIC was assessed by *in situ* electroporation at the indicated confluences (see Methods, Figure 8). Quantitation was achieved by dividing the number of cells into which the dye had transferred through gap junctions (denoted by dots, Figure 1B and 2A), by the number of cells at the edge of the electroporated area (denoted by stars). Numbers are averages ±SEM of at least three experiments, where transfer from more than 200 cells was examined.

N/A: Not applicable.

We next examined the levels of Cx43, a widely expressed gap junction protein, at different cell densities. Cells were plated in plastic petri dishes at a confluence of 80% and at different times up to 5 days post confluence, total protein extracts were probed for Cx43 by Western blotting. As shown in Figure 1C, cell density caused a dramatic increase in Cx43 levels, which plateaued at ~3 days post-confluence (lane 1 *vs* 5).

### GJIC and connexin-43 in NSCLC lines and freshly explanted tumor cells

**Figure 2:**
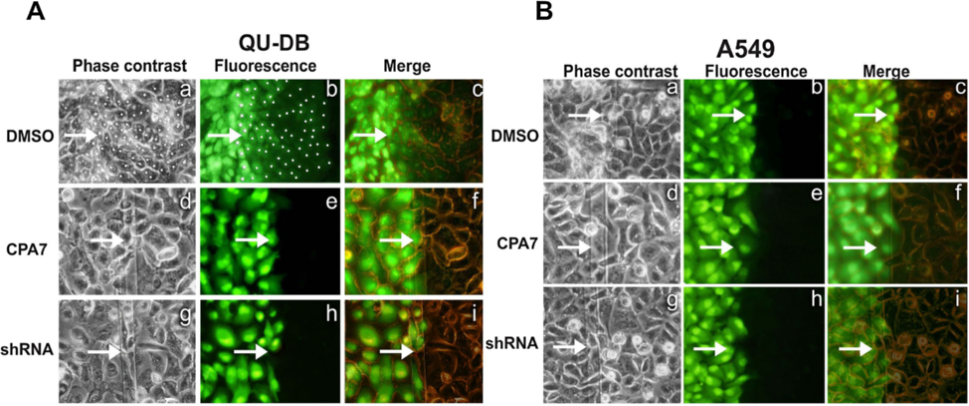
**A. Stat3 downregulation eliminates gap junctional permeability in human lung carcinoma QU-DB cells.** QU-DB cells were plated in electroporation chambers and subjected to a pulse in the presence of Lucifer yellow, following treatment with the DMSO carrier alone (***a-c***), or CPA7 (***d-f***), or infection with the sh-Stat3 lentiviral vector (***g-i***) (see Methods, Figure 8). After washing away the unincorporated dye, cells from the same field were photographed under fluorescence (***b, e, h***) or phase contrast (***a, d, g***) illumination. Cells at the edge of the conductive area which were loaded with LY through electroporation were marked with a star, and cells at the non-electroporated area which received LY through gap junctions were marked with a dot [4]. Arrows point to the edge of the electroporated area. ***c, f, i***: Overlay of phase-contrast and fluorescence. Magnification: 240 x. Note the extensive gap junctional communication in (***b***). **B. Stat3 downregulation does not increase gap junctional permeability in human lung carcinoma A549 cells.** Same as above, A549 cells. Note the absence of GJIC, even after Stat3 downregulation (***e, h***).

**Figure 3:**
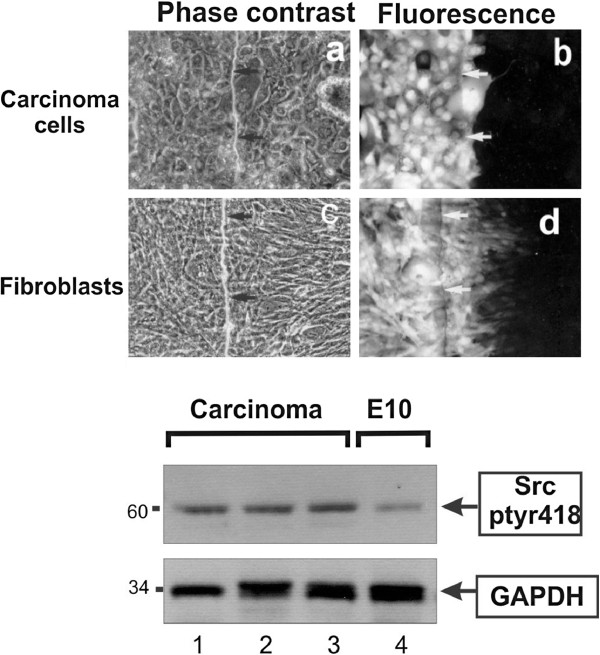
**Primary lung carcinoma cells display low gap junctional, intercellular communication. a** and **b**: Cells cultured from a freshly explanted lung tumor specimen were grown in electroporation chambers and Lucifer yellow introduced with an electrical pulse (Figure 7, [42]). Arrows point to the edge of the electroporated area. Note the absence of gap junctional communication. Magnification: 240x. **c** and **d**: Following growth of the cells for 10 weeks, fibroblasts present in the original cell suspension predominated. They were plated in electroporation chambers and Lucifer yellow introduced with an electrical pulse. Note the extensive communication through gap junctions. **Lower panel** Extracts of cells cultured from a moderately differentiated adenosquamous carcinoma, a poorly differentiated adenocarcinoma, and adenocarcinoma, respectively (lanes 1–3), or E10 cells (lane 4), were probed for Src-ptyr418 or GAPDH as a loading control, as indicated.

In light of the above findings, we examined GJIC levels at different densities up to 4 days post-confluence in a panel of human lung cancer lines [18]. Two NSCLC lines, QU-DB (Figure 2A, ***a-c***) and SK-LuCi6 (Table 1,A) displayed extensive GJIC at their peak density, while five NSCLC lines had very low GJIC (e.g. A549, Figure 2B, ***a-c,*** and Table 1,B). In addition, primary cells explanted and cultured from a moderately differentiated adenosquamous carcinoma (Figure 3, ***a-b***), a poorly differentiated adenocarcinoma, and an adenocarcinoma (Table 2) had no GJIC.

**Table 2:** **GJIC in primary lung carcinoma cells**^**α**^

	** Cells**^**β**^	**GJIC**^**α**^
**Adenosquamous carcinoma, moderately differentiated**	**carcinoma cells**	0.1 ±0.1
	**fibroblasts**	5.8±1.2
**Adenocarcinoma, poorly differentiated**	**carcinoma cells**	0.1 ±0.1
**Adenocarcinoma**	**carcinoma cells**	0.1 ±0.1

^**α**^Immediately after surgery, cells were placed in culture and GJIC examined (see Methods, Figure 7). After 8-10 weeks in culture, most of the tumor cells had died while the fibroblasts present in the initial suspension predominated. These cells did not express cytokeratins, contrary to tumor cells [18]. The fibroblasts shown were derived from the moderately differentiated adenosquamous carcinoma tumor above (Figure 3, *c-d*). GJIC was examined as in Table 1, at 3 days after confluence.

**Figure 4:**
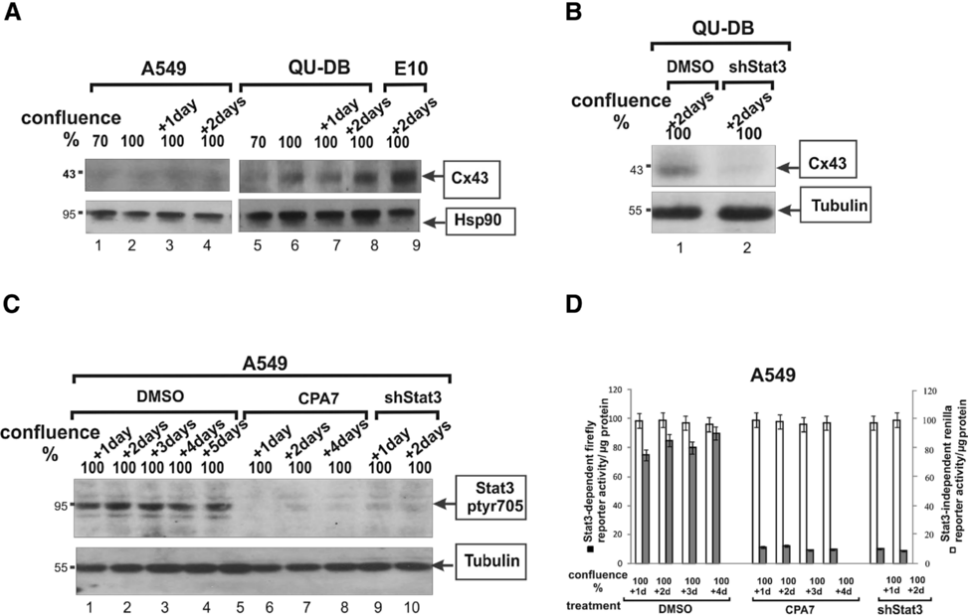
**A: Cell density causes a dramatic increase in Cx43 levels in QU-DB cells.** QU-DB (lanes 5-8) or A549 (lanes 1-4) or nontransformed E10 (lane 9) cells were grown to different densities as indicated and extracts probed for Cx43 or Hsp90 as a loading control. Note the absence of Cx43 in A549 cells and the increase in Cx43 with density in QUDB. **B: Stat3 knockdown reduces Cx43 levels.** QU-DB cells infected with the lentiviral vector carrying the Stat3-specific shRNA (lane 2) or not infected (lane 1) were grown to 2 days post-confluence and lysates probed for Cx43 or Hsp90 as a loading control. **C: CPA7 or Stat3-knockdown with shRNA reduce Stat3-ptyr705 levels in A549 cells.** A549 cells were grown to increasing densities and treated with the Stat3 inhibitor, CPA7 (lanes 6-8) or the DMSO carrier (lanes 1-5) for 15 hrs and cell extracts probed for Stat3-ptyr705 or tubulin as a loading control. Parallel cultures were infected with a vector expressing a Stat3-specific, shRNA [37], and cell extracts from stable lines produced were probed as above. **D: CPA7 or Stat3-knockdown with shRNA reduce Stat3 transcriptional activity in A549 cells.** A549 cells were transfected with a plasmid expressing a firefly luciferase gene under control of a Stat3-responsive promotor (▪) and a Stat3-independent promotor driving a *Renilla* luciferase gene (□) (see Methods). After transfection, cells were plated to different densities and treated with CPA7 or the DMSO carrier alone for 24 hrs, at which time firefly and *Renilla* luciferase activities were determined. Parallel cultures expressing the sh-Stat3 construct were transfected with the plasmids and firefly and *Renilla* luciferase activities determined.

Examination of Cx43 levels showed that QU-DB cells had levels similar to E10, which increased dramatically with cell density, while Cx43 levels in A549 cells were almost undetectable, at any cell density (Figure 4A). SK-LuCi6 cells had levels similar to QU-DB, while all other NSCLC lines examined had very low Cx43 levels at all densities tested (not shown). The above data taken together indicate that, besides nontransformed epithelial cells, cell density causes a dramatic increase in GJIC and Cx43 protein levels in two lung carcinoma lines which display extensive GJIC. Nevertheless, the majority of lung cancer lines examined (5/7) had very low or no detectable gap junctional communication, even at high cell densities (Table 1,B).

### Src activity and GJIC suppression in NSCLC lines

**Figure 5:**
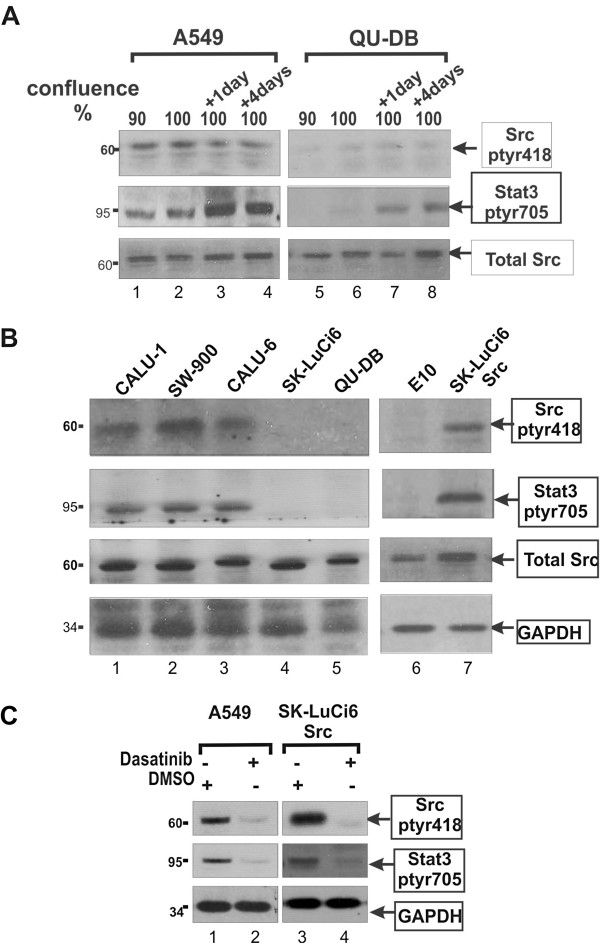
**A: A549 cells have high Src-ptyr418 levels.** QU-DB (lanes 5-8) or A549 (lanes 1-4) cells were grown to different densities as indicated and extracts probed for Src-ptyr418, Stat3-ptyr705 or total Src. Note the low levels of Src-ptyr418 in QU-DB cells. **B: Src-ptyr418 and Stat3-ptyr705 in NSCLC lines.** The indicated cell lines were grown to 50% confluence and extracts probed for Src-ptyr418, Stat3-ptyr705, total Src or GAPDH as a loading control. **C: Dasatinib reduces Stat3-ptyr705 levels in A459 cells:** A549 or SK-LuCi6-*Src* cells were grown to subconfluence and treated with the Src-selective inhibitor, Dasatinib (1μM) or the DMSO carrier alone and cell extracts probed for Src-ptyr418, Stat3-ptyr705 or GAPDH as a loading control, as indicated.

We next examined Src-tyr418 phosphorylation, as an indication of Src activity. As shown in Figure 5A and C, A549 cells displayed high Src-ptyr418 levels, similar to the levels in SK-LuCi6 or E10 cells expressing activated Src by retroviral transduction (lines SK-LuCi6-***Src***, E10-***Src***, respectively, Figure 5C, lanes 1 *vs* 3 and Table 1,B), while Src-ptyr418 levels in QU-DB cells were low (Figure 5A, lanes 5-8), similar to E10 (Figure 5B, lanes 5 and 6). Lines CALU-1, SW-900, CALU-6 and SK-Lu1 had Src-ptyr418 levels comparable to SK-LuCi6-***Src*** (Figure 5B, lanes 1-3 *vs* 7 and Table 1,B), while SK-LuCi6 had low levels, similar to QU-DB (Figure 5B, lanes 4-5). Examination of gap junctional communication revealed that five lines with high Src-ptyr418 (A549, CALU-1, SW-900, CALU-6, LuCi-1) had very low or no detectable GJIC (Figure 2B, ***a-c*** and Table 1,B). In addition, Src expression in SK-LuCi6 or E10 cells eliminated junctional permeability (E10-***Src*** and SK-LuCi6-***Src***, Table 1, B), in agreement with the known Src effect of GJIC suppression. Conversely, the two lines with low Src-ptyr418 levels (QU-DB and SK-LuCi6), had high GJIC, especially at high densities (Figure 2A and Table 1, A). Primary cells from the three tumor specimens were found to have higher Src activity than the E10, consistent with previous results from biopsy tissues (Figure 3, bottom panel). Taken together, these data point to an inverse relationship between Src activity levels and GJIC in these NSCLC lines.

### Stat3 is a positive regulator of GJIC in NSCLC lines

Stat3 is a prominent effector of the non-receptor tyrosine kinase Src [24]. However, Stat3 can be activated by cytokine and membrane tyrosine kinase receptors, which can act in a Src-independent manner [11]. Therefore, to assess the specific contribution of Src to Stat3 activation in the lung cancer lines, we at first examined the correlation between Src-ptyr418 and Stat3-ptyr705 levels. As shown before for a number of cell types (reviewed in [17]), high cell density caused an increase in Stat3-ptyr705 levels in all lines (e.g. Figure 5A, lanes 1-4 and 5-8), therefore Stat3-ptyr705 levels were assessed at a confluence of 50% for this experiment (see Methods). The results showed elevated Stat3-ptyr705 levels in the five lines with high Src-ptyr418 at all cell densities, comparable to SK-LuCi6-***Src*** cells (e.g. A549 *vs* QU-DB, Figure 5A, lanes 1-4 *vs* 5-8 and Figure 5B and Table 1,B). At the same time, QU-DB and SK-LuCi6 cells had low levels of both Src-ptyr418 and Stat3-ptyr705 (Figure 5B). The above data point to a correlation between Src and Stat3 activity levels in the NSCLC lines. We next examined the effect of Src inhibition upon Stat3-ptyr705 in the lines found to have high Src-ptyr418. The results showed that treatment with the Src inhibitor Dasatinib caused a dramatic reduction in Stat3-ptyr705 (e.g. line A549, Figure 5C, and Additional file 1: Additional data, Table Add-I). Similar results were obtained with the PD180970 and SU6656 Src inhibitors (see Methods). These findings indicate that Src may, in fact, be an important Stat3 activator in these cells.

The effect of Stat3 inhibition upon GJIC in the 5 lines with high Src activity was examined next. As shown in Figure 3C and D treatment with the Stat3 inhibitor, CPA7 for 15 hrs [25], or knockdown with a Stat3-specific, shRNA, essentially eliminated Stat3, tyr705 phosphorylation and activity in A549 cells. However, CPA7 treatment (Figure 2B, ***d-f***), or Stat3 knockdown (Figure 2B, ***g-i***) did not increase junctional permeability in A549 cells. Similar results were obtained with SK-Lu1, CALU-1, SW-900 and CALU-6 lines (Table 1,B). The above data taken together indicate that the high Stat3 activity, which could be, at least in part, due to high Src activity in these lines, cannot be responsible for the lack of junctional communication in the lung carcinoma lines examined.

Since the lung cancer lines might express other oncogenes besides Src, we examined the role of Stat3 in the Src-triggered GJIC suppression specifically, using the Src-transduced, SK-LuCi6-***Src*** line. As expected, Src expression disrupted gap junctional permeability. Interestingly, subsequent Stat3 inhibition with CPA7 or shRNA did not restore GJIC (Table 1,B). Taken together, the above findings indicate that Stat3 cannot be part of a pathway leading to Src-induced, gap junction closure in SK-LuCi6-***Src*** cells.

We then examined the possibility that Stat3 might play a ***positive*** role in the maintenance of gap junctional permeability, by assessing the effect of Stat3 inhibition upon GJIC levels in QU-DB cells which have low Src activity and extensive GJIC. As shown in Figure 2A (***d-f***), Stat3 downregulation through CPA7 treatment essentially ***abolished*** GJIC in QU-DB cells. Reduction of Stat3 levels through infection with the sh-Stat3 lentivirus vector gave similar results (Figure 2A, ***g-i***). Similarly, Stat3 downregulation in SK-LuCi6 or E10 cells caused a dramatic decrease in GJIC (Table 1,A). Conversely, expression of the constitutively active form of Stat3, Stat3C [26], increased the already extensive gap junctional communication in SK-LuCi6 cells (Table 1,A).

**Figure 6:**
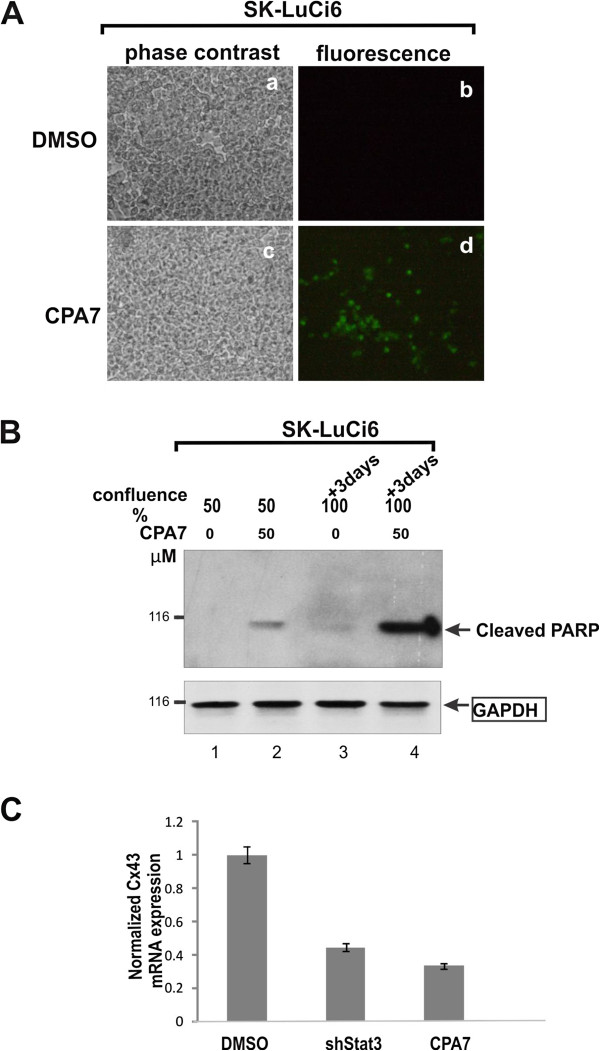
**A, B: Stat3 inhibition induces apoptosis A:** SK-LuCi6 cells without (***a,b***) or with (***c,d***) CPA7 treatment were fixed and stained for TUNEL using FITC-coupled, nucleotide triphosphates (see Methods). **B**: Extracts from SK-LuCi6 cells grown to 50% confluence or 3 days post-confluence, without (lanes 1,3) or with (lanes 2,4) treatment with 50 μM CPA7 for 15 hrs as indicated, were probed for cleaved PARP, with Hsp90 as a loading control (lower panel). **C: Stat3 inhibition reduces Cx43 mRNA.** Total RNA from SK-LuCi6 cells treated with the CPA7, Stat3 inhibitor, or the DMSO carrier alone, or stably expressing shStat3 as indicated, was subjected to quantitative RT-PCR analysis (see Methods).

Examination of Cx43 levels following sh-Stat3 expression revealed a dramatic reduction (Figure 4B), indicating that Stat3 is required for the maintenance of Cx43 protein levels. TUNEL staining revealed that Stat3 inhibition by CPA7 treatment caused an increase in apoptosis in SK-LuCi6 cells (Figure 6A). In addition, CPA7 treatment caused an increase in PARP cleavage in these cells, even at a confluence of 50% (Figure 6B, lane 2). At 3 days post confluence, the time of GJIC examination, PARP cleavage was greater (lane 4), in agreement with previous results indicating that Stat3 inhibition causes apoptosis which is more pronounced in confluent cultures [27]. This finding hints at a link between GJIC reduction and apoptosis induced by Stat3 inhibition.

We next examined whether Stat3 inhibition might also affect Cx43 mRNA levels, through quatitative RT-PCR analysis [28]. The results showed that Stat3 inhibition by CPA7 treatment, or downregulation through shRNA expression brought about a substantial reduction in Cx43 mRNA levels, indicating an effect of Stat3 upon Cx43 gene transcription as well. In any event, taken together, our data reveal that, rather than increasing junctional permeability as might have been expected based on the well documented ability of Stat3 to act as a Src effector, Stat3 inhibition eliminates GJIC, indicating that Stat3 activity is actually ***required*** for gap junction function in two cultured lung carcinoma lines which display extensive GJIC.

## Discussion

Extensive data from our group and others demonstrated that oncogenes such as mT, Src or Ras can suppress gap junctional, intercellular communication [3,6]. Moreover, it was shown that lower levels of these gene products were sufficient to eliminate gap junction function than the levels necessary for full transformation [4,29], indicating that a decrease in GJIC may be an early event in neoplastic conversion. In this communication we used an improved procedure to examine GJIC in lung cancer lines as well as in primary lung tumor cells. All cell lines had been established from NSCLC tumors which were known to be metastatic [18], except QU-DB, which was derived from a patient that was a long term survivor [30]. Our results reveal that GJIC was low in the majority of cases, except in the QU-DB and SK-LuCi6 lines. Assuming that the establishment process did not bring about an ***increase*** in GJIC, the existence of extensive GJIC in line SK-LuCi6 which was established from a rapidly metastatic tumor [31] indicates that intercellular communication does not necessarily inhibit metastasis; other factors may supercede potential growth inhibitory effects of intercellular communication and may be responsible for tumor growth and metastasis.

We next examined the mechanism of GJIC suppression by assessing the role of Src and its effector Stat3. Our results revealed an inverse relationship between Src-tyr418 phosphorylation levels and GJIC in a number of lines. Since Src is known to suppress gap junctional communication in cultured cells such as rodent fibroblasts and epithelial cells, it is tempting to speculate that Src may be responsible, at least in part, for gap junction closure in these lines. However, repeated attempts to reinstate GJIC by reducing Src activity levels through treatment with the Src kinase family-selective, pharmacological inhibitors Dasatinib, PD180970 or SU6656, or infection with Adenoviral vectors expressing a Src dominant-negative mutant or c-Src kinase [15] in A549 cells which have high Src-ptyr418 were unsuccessful (not shown). Possibly other oncoproteins besides Src, or other factors may be important contributors to GJIC suppression in these lines. Alternatively, since low levels of activated Src were previously shown to be sufficient for GJIC suppression in mouse fibroblasts [3,4], the possibility that the residual Src activity in treated cells might be sufficient to interrupt gap junctional communication cannot be excluded. Dasatinib treatment of SK-LuCi6-***Src*** cells did cause a partial restoration of GJIC, although the high levels of SK-LuCi6 were not attained, possibly due to the high Src activity levels in this line.

We also examined GJIC in freshly explanted, primary cells from 3 NSCLC specimens. Since the senescence process can reduce GJIC [32], cells were plated in electroporation chambers immediately after surgery at densities of ~80%, so that they would reach confluence within 1–2 days, and GJIC examined every day for up to 10 days. No gap junctional communication was ever detected in any of the preparations, although fibroblasts from the same tissue had extensive GJIC (Figure 3, ***c-d***). Src-418 levels were relatively high in cells from all three tumor specimens, indicating that Src may have played a role in GJIC suppression. However, the possibility that the initiation of the senescence process even a day after surgery may have affected GJIC cannot be excluded.

### Stat3 does not transmit Src signals to gap junction closure

Several signal transducers besides Stat3 are known to be downstream effectors of the Src kinase such as Ras/Raf/Erk, PI3k/Akt, the Crk-associated substrate (Cas) and others [33]. Constitutively active Ras is neoplastically transforming and can suppress GJIC [6,29]. Examination of the mechanism of Src-mediated, GJIC suppression previously indicated that inhibition of Ras in Src-transformed, rat fibroblasts reinstated gap junctional communication [19]. Conversely, mT expression in Ras-deficient cells did not suppress GJIC [34]. These data taken together underline the importance of the Ras pathway in GJIC reduction by activated Src. It was also shown later that Cas is required for the Src-induced, reduction in gap junctional communication [35]. In sharp contrast, our present data with Src-transduced, SK-LuCi6-***Src*** cells demonstrate that Stat3 inhibition does not restore GJIC, indicating that a role of Stat3 in the Src-induced, GJIC suppression in these cells is unlikely, despite the fact that constitutively active Stat3 can act as an oncogene and transform established lines [36].

### Stat3 plays a *positive* role in gap junctional communication

The fact that cell density upregulates Stat3 concomitant with an increase in both Cx43 and GJIC prompted us to explore a potential positive role of Stat3 upon GJIC. Interestingly, Stat3 inhibition in two NSCLC lines which exhibit extensive junctional communication (QU-DB, SK-LuCi6) abolished GJIC, indicating that Stat3 does in fact play a positive role in the maintenance of gap junction function. This conclusion is in agreement with a previous report indicating that Stat3 inhibition eliminated GJIC in nontransformed rat liver epithelial cells as well [37].

Results from a number of labs demonstrated that Stat3 activates a number of anti-apoptotic genes, such as BcL-xL, Mcl1 and Akt1 [11]. Global induction of apoptosis with etoposide, cycloheximide or puromycin was shown to lead to a loss of cell coupling, probably due to caspase-3-mediated degradation of Cx43, in primary bovine lens epithelial and mouse NIH3T3 fibroblasts [38]. Interestingly, we previously demonstrated that Stat3 inhibition in cells transformed by Src or the Large Tumor antigen of Simian Virus 40 leads to apoptosis [15,39], possibly due to activation of the transcription factor E2F family, potent apoptosis inducers, by these oncogenes. Therefore, apoptosis induced by Stat3 downregulation in cells with high Src may have triggered gap junction closure.

We previously demonstrated that while Stat3 inhibition in sparsely growing, normal mouse fibroblasts causes growth retardation, at high densities, such as needed for optimal gap junction formation, Stat3 inhibition leads to apoptosis [27]. Therefore, apoptosis induction through a reduction in Stat3 levels or activity could explain the dramatic reduction in Cx43 and GJIC upon Stat3 pharmacological or genetic inhibition, in lines with low Src activity. Still, our results also demonstrate a substantial reduction in Cx43 mRNA levels upon Stat3 inhibition, pointing to a transcriptional effect of Stat3 upon the Cx43 promotor in these NSCLC lines, as previously demonstrated in other cell types [28,40,41].

## Conclusions

Our results demonstrate that Stat3 is not transmitting Src signals leading to gap junction closure in the NSCLC cell lines examined. In the contrary, although Stat3 is generally growth promoting and in an activated form it can act as an oncogene, we show for the first time that Stat3 is actually ***required*** for gap junctional communication both in normal epithelial cells and in certain tumor cell lines that retain GJIC. This novel role of Stat3 in gap junction function may be an important regulatory step in progression of tumours that exploit such a pathway.

## Methods

### Examination of gap junctional communication

**Figure 7:**
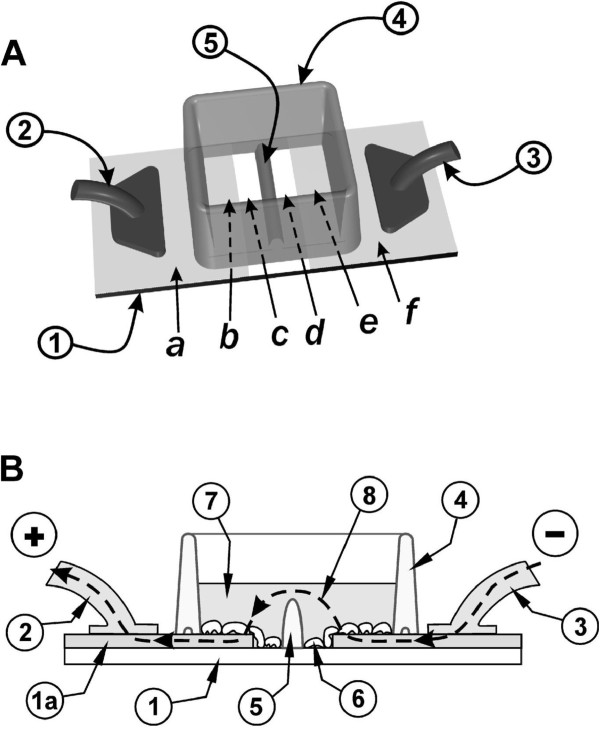
**Electroporation on two co-planar ITO electrodes, formed by removing the ITO coating by chemical etching A: Top view.** Cells were grown on an ITO-coated slide from which the coating was removed in a strip as shown. The two conductive sides (***a, f***), serving as electrodes, were connected to the positive and negative poles of the pulse generator (**2**) and (**3**). A nonconductive barrier (**5**) divides the strip of bare glass in half and separates the chamber into two sections. **B: Side view.** The slide with the cells growing on the ITO coated and the bare glass regions is shown. When electroporation buffer is added to the chamber to a level above the height of the barrier (**5**) then an electrical path between the electrodes (***e*** and ***b***) is formed. Note that the ITO layer (1***a***) is shown with dramatically exaggerated thickness for clarity, although its actual thickness is much less than the thickness of the cells (from [42]).

To examine gap junctional communication by *in situ* electroporation, it is important to be able to reliably distinguish cells that were loaded with Lucifer yellow directly by electroporation, from cells that received the dye from neighbouring cells by diffusion through gap junctions. This was achieved using a slide where a 3 mm-wide strip of ITO had been removed by etching with acids, leaving two co-planar electrodes, supported by the same glass slide substrate (Figure 7) [42].

**Figure 8:**
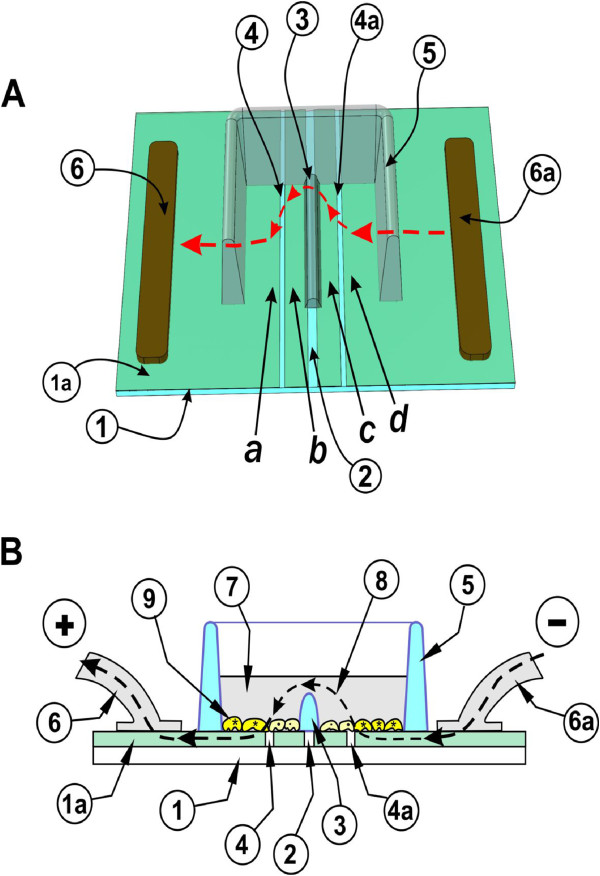
**Electroporation on two co-planar ITO electrodes, formed by removing the ITO coating by etching with Lasers. A:** Top view. Cells are grown on a glass slide (**1**), coated with ITO (**1*a***). The coating is laser-etched in a straight line in the middle (**2**), essentially forming two electrodes. A dam of Teflon (**3**) is used to divert the current upwards, thus creating a sharp transition in electric field intensity. A plastic chamber is bonded onto the slide, to form a container for the cells and LY (**5**). To provide areas where the cells are not electroporated, the ITO was also removed in two parallel lines [(**4**), (**4a**)]. Current from a pulse generator flows inwards from each contact point (**6** and **6*a***) to area (***d***) then over the barrier [(**3**), arrowheads] to the other side, electroporating cells in area (***a***). For clarity, the front part of the chamber is removed. **B: Side view.** The slide (**1**) with the cells growing on the ITO coated [(**1*a***), light green] and etched, bare glass regions is shown. When electroporation medium (**7**) is added to the chamber to a level above the height of the dam (**3**) then an electrical path (arrowheads) between the electrodes (**6**) and (**6*a***) and the cells (**9**) growing in this area is formed. Note that the size of the cells and the ITO layer (**1*a***) are shown exaggerated for clarity although the actual thickness of the ITO (800 Å) is much lower than the thickness of the cells.

In a further improvement (Figure 8), the coating was removed from the glass surface in ~20 μm wide lines, to define electrode and non-conducting regions. Etching was done using a laser beam, so that the nonconductive glass underneath is exposed. It was important to ensure that only the 800Å coating was removed, without affecting the glass, so that cell growth would be unaffected across the line. This was achieved with a UV laser operating at a 355 nm wavelength using approximately 1 Watt of output power with 60% of the energy delivered to the surface of the glass. The beam was manipulated by mirrors on a pair of galvanometers to produce the desired pattern.

To form the two electrodes, the coating was removed in a straight line in the middle (**2**). A dam of nonconductive plastic (**3)** was bonded onto this line, to divert the current upwards, thus creating a sharp transition in electric field intensity between electroporated and non-electroporated sections. To provide areas where the cells are not electroporated, the ITO was also removed in two parallel lines [(**4) and** (**4*a***)]. A plastic chamber was bonded onto the slide, to form a container for the cells and electroporation solutions (**5)**. Current flows inwards from each contact point (**6** and **6*a***), via a conductive highway under the well (**5)** electroporating cells in area (***d***) then over the barrier [(**8)**, arrowheads] to the other side, in area (***a***). In this configuration, cells which acquired LY by electroporation [growing in (***a***) and (***d***)] and cells into which LY traveled through gap junctions [ (***b***) and (***c***)] both grow on ITO, separated only by a laser-etched line of ~20 μm. Extensive experimentation showed that in this setup the electroporation intensity is uniform across the electroporated area (see Figure 1B and Figure 2).

Cells were plated in the chamber and when they reached the appropriate density (90% confluence, to 5 days post-confluence), the growth medium was replaced with Calcium-free DMEM supplemented with 5 mg/ml Lucifer yellow (**7)**. The slide/chamber was placed into a holder where electrical contacts were established and a set of electrical pulses delivered to the cells. Extensive experimentation indicated that 10 pulse pairs, each pulse of 18 Volts peak value, 100 μs length and spaced 0.5 seconds apart, with one of each pair having a polarity opposite to that of its partner gave optimal results. Following a 5 min incubation at 37°C, the unincorporated dye was washed away with Calcium-free DMEM supplemented with 10% dialysed fetal calf serum and cells observed and photographed under fluorescence and phase contrast illumination. Communication is expressed as the number of cells into which the dye has transferred per cell loaded with the dye by electroporation at the edge of the electroporated area. All experiments were conducted at least three times, with at least 5 slides each time, and the results are presented as average GJIC±SEM where the transfer from at least 200 cells is assessed.

The equipment (*Insitu* Porator) was supplied by Cell Projects Ltd UK.

### Cell lines, culture techniques and Stat3 activity measurement

All cells were grown in DMEM with 10% fetal calf serum. Extra care was taken to ensure that cell seeding was uniform, by passing cells at subconfluence, when cell to cell adhesion was low. Confluence was estimated visually and quantitated by imaging analysis of live cells under phase contrast [14]. To ensure that the growth medium was not depleted of nutrients, it was changed every day.

Cells were cultured from surgically explanted tumors as previously described [18].

Stat3C and activated Src were expressed in SK-LuCi-6 cells through infection with the culture supernatant from a Phoenix amphotropic packaging line transfected with a pBabe-puro-Stat3C plasmid [26]. shStat3 was expressed by retroviral vector infection as described [16].

Stat3 transcriptional activity was measured as described, by transient transfection of the p*Luc*TKS3 construct [24]. As a control, cells were co-transfected with the reporter pRLSRE, which contains two copies of the serum response element (SRE) of the c-fos promoter, subcloned into the *Renilla* luciferase reporter, pRL-null (Promega) [15]. Following transfection, cells were plated to different densities and luciferase activity determined.

For Stat3 immunostaining, SK-LuCi6 cells were fixed with 4% paraformaldehyde, permeabilized in 0.2% Triton-X100 and probed with a Stat3 antibody (Cell Signalling, #9132 diluted at 1:100) followed by AlexaFluor-coupled, goat anti-rabbit IgG (Invitrogen #A11008, used at 1:400).

### Inhibitors

Stat3 was inactivated using two approaches: **(1).** Treatment with 50 μM CPA7 [PtCl_3_(NO_2_)(NH_3_)_2_[25] overnight, or **(2)**. Expression of shRNA, delivered with a lentivirus vector as described [37]. Jak inhibitor-1 was from EMD Biosciences (5 μM [16]).

Src was inactivated using 3 pharmacological inhibitors: Dasatinib (0.5 or 1 μM, up to 72h), PD180970 (0.2 μM with redosing every 12h for a total of 24h), or SU6656 (5μM for 24h) [15].

### Western blotting

It was conducted on proteins extracted from cell pellets [43], using antibodies to Cx43 (Cell Signalling, #3512, used at a 1:500 dilution), Stat3-ptyr705 (Cell Signalling, #9131, 1:1,000), Src-ptyr418 (Invitrogen, #44-660G , 1:1,000) or total Src (rabbit monoclonal 36D10, Cell Signalling, #2109, 1:1,000), followed by secondary antibodies and ECL reagents (Biosource). Alpha-Tubulin (Cell Signalling #2125, 1:5,000), GAPDH (BD Transduction, #14C10, 1:5,000) or Hsp90 (Assay designs, #SPA-830, 1:5,000) served as loading controls.

### qRT-PCR

SK-LuCi6 cells were treated with CPA7 for 15 hrs. The RNeasy Mini Kit (Qiagen, Hilden Germany, cat. #74104) was used for the purification of RNA. cDNA synthesis from 1μg of total RNA was performed using the iScript cDNA synthesis kit (Bio-RAD Laboratories, Hercules, CA). qRT-PCR was performed using iQ SYBR Green Supermix (Bio-RAD Laboratories, Hercules, CA) and 20μM primer. Primer sequences were [28]:

**Cx43: Forward:** 5^′^-GCCTGAACTTGCCTTTTCAT-3^′^,

Reverse: 5^′^- CTCCAGTCACCCATGTTGC-3^′^, [28] to generate a product of 500 bp [40].

As internal reference genes we used GAPDH: (forward: 5^′^-AATGCATCCTGCACCACCAA-3^′^, Reverse: 5^′^-GTAGCCATATTCATTGTCATA-3^′^) [40] and 18S RNA [16]. mRNA from SK-LuCi6 cells where Stat3 was downregulated with sh-Stat3 was analysed in a similar manner. Results from 3 independent experiments, each conducted in triplicate were averaged out and SEM calculated.

## Abbreviations

Cx43: Connexin-43; GJIC: Gap junctional, intercellular communication; NSCLC: Non-small cell lung cancer.

## Competing interests

The corresponding author received a royalty from PARTEQ, the intellectual property arm of Queen’s University, for a patent where she is a co-inventor of the apparatus used in this work. PARTEQ had no involvement whatsoever in the content of this paper.

## Authors’ contributions

MG did the bulk of the benchwork. SG and RA conducted some of the initial experiments. Aaron Trotman-Grant did the Stat3C experiments. ET obtained the results on the primary lung carcinoma cells. LR conceived of the study, designed and coordinated it and drafted the manuscript. All authors read and approve of the manuscript.

## Authors’ information

MG is a postdoctoral fellow, funded by the US Army breast cancer program. RA and ET were graduate students. ATG is currently doing a project. LR is a professor at Queen’s University, Kingston, Canada.

## Pre-publication history

The pre-publication history for this paper can be accessed here:

http://www.biomedcentral.com/1471-2407/12/605/prepub

## Supplementary Material

Additional file 1 Additional data. Click here for file

## References

[B1] Vinken M Vanhaecke T Papeleu P Snykers S Henkens T Rogiers V Connexins and their channels in cell growth and cell death Cell Signal 2006 18 592 600 10.1016/j.cellsig.2005.08.012 16183253

[B2] Lin R Martyn KD Guyette CV Lau AF Warn-Cramer BJ v-Src tyrosine phosphorylation of connexin43: regulation of gap junction communication and effects on cell transformation Cell Commun Adhes 2006 13 199 216 10.1080/15419060600848516 16916748 PMC2712291

[B3] Azarnia R Loewenstein WR Polyomavirus middle t antigen downregulates junctional cell-to-cell communication Mol Cell Biol 1987 7 946 950 2434836 10.1128/mcb.7.2.946-950.1987 PMC365156

[B4] Raptis L Brownell HL Firth KL MacKenzie LW A novel technique for the study of intercellular, junctional communication; electroporation of adherent cells on a partly conductive slide DNA Cell Biol 1994 13 963 975 10.1089/dna.1994.13.963 7917017

[B5] Grammatikakis N Vultur A Ramana CV Siganou A Schweinfest CW Raptis L The role of Hsp90N, a new member of the Hsp90 family, in signal transduction and neoplastic transformation J Biol Chem 2002 277 8312 8320 10.1074/jbc.M109200200 11751906

[B6] Brownell HL Narsimhan R Corbley MJ Mann VM Whitfield JF Raptis L Ras is involved in gap junction closure in mouse fibroblasts or preadipocytes but not in differentiated adipocytes DNA Cell Biol 1996 15 443 451 10.1089/dna.1996.15.443 8672240

[B7] Atkinson MM Sheridan JD Altered junctional permeability between cells transformed by v-ras, v-mos, or v-src Am J Physiol 1988 255 C674 C683 3056028 10.1152/ajpcell.1988.255.5.C674

[B8] Pahujaa M Anikin M Goldberg GS Phosphorylation of connexin43 induced by Src: regulation of gap junctional communication between transformed cells Exp Cell Res 2007 313 4083 4090 10.1016/j.yexcr.2007.09.010 17956757

[B9] Masaki T Igarashi K Tokuda M Yukimasa S Han F Jin YJ pp60c-src activation in lung adenocarcinoma Eur J Cancer 2003 39 1447 1455 10.1016/S0959-8049(03)00276-4 12826049

[B10] Zhang J Kalyankrishna S Wislez M Thilaganathan N Saigal B Wei W SRC-family kinases are activated in non-small cell lung cancer and promote the survival of epidermal growth factor receptor-dependent cell lines Am J Pathol 2007 170 366 376 10.2353/ajpath.2007.060706 17200208 PMC1762707

[B11] Yu H Pardoll D Jove R STATs in cancer inflammation and immunity: a leading role for STAT3 Nat Rev Cancer 2009 9 798 809 10.1038/nrc2734 19851315 PMC4856025

[B12] Song L Turkson J Karras JG Jove R Haura EB Activation of Stat3 by receptor tyrosine kinases and cytokines regulates survival in human non-small cell carcinoma cells Oncogene 2003 22 4150 4165 10.1038/sj.onc.1206479 12833138

[B13] Byers LA Sen B Saigal B Diao L Wang J Nanjundan M Reciprocal regulation of c-Src and STAT3 in non-small cell lung cancer Clin Cancer Res 2009 15 6852 6861 10.1158/1078-0432.CCR-09-0767 19861436 PMC2935176

[B14] Vultur A Cao J Arulanandam R Turkson J Jove R Greer P Cell to cell adhesion modulates Stat3 activity in normal and breast carcinoma cells Oncogene 2004 23 2600 2616 10.1038/sj.onc.1207378 15007380

[B15] Vultur A Arulanandam R Turkson J Niu G Jove R Raptis L Stat3 is required for full neoplastic transformation by the Simian Virus 40 Large Tumor antigen Mol Biol Cell 2005 16 3832 3846 10.1091/mbc.E04-12-1104 15917293 PMC1182320

[B16] Arulanandam R Vultur A Cao J Carefoot E Truesdell P Elliott B Cadherin-cadherin engagement promotes survival via Rac/Cdc42 and Stat3 Mol Cancer Res 2009 17 1310 1327 19671682 10.1158/1541-7786.MCR-08-0469

[B17] Raptis L Arulanandam R Vultur A Geletu M Chevalier S Feracci H Beyond structure, to survival: Stat3 activation by cadherin engagement Biochem Cell Biol 2009 87 835 843 10.1139/O09-061 19935869

[B18] Tomai E Brownell HL Tufescu T Reid K Raptis L Gap junctional communication in lung carcinoma cells Lung Cancer 1999 23 223 231 10.1016/S0169-5002(99)00016-1 10413198

[B19] Ito S Ito Y Senga T Hattori S Matsuo S Hamaguchi M v-Src requires Ras signaling for the suppression of gap junctional intercellular communication Oncogene 2006 25 2420 2424 10.1038/sj.onc.1209263 16301992

[B20] Wei CJ Francis R Xu X Lo CW Connexin43 associated with an N-cadherin-containing multiprotein complex is required for gap junction formation in NIH3T3 cells J Biol Chem 2005 280 19925 19936 10.1074/jbc.M412921200 15741167

[B21] Frenzel EM Johnson RG Gap junction formation between cultured embryonic lens cells is inhibited by antibody to N-cadherin Dev Biol 1996 179 1 16 10.1006/dbio.1996.0237 8873750

[B22] Vultur A Tomai E Peebles K Malkinson AM Grammatikakis N Forkert PG Gap junctional, intercellular communication in cells from urethane-induced tumors in A/J mice DNA Cell Biol 2003 22 33 40 10.1089/104454903321112479 12590735

[B23] Schiller J Sabatini L Bittner G Pinkerman C Mayotte J Levitt M Phenotypic, molecular and genetic-characterization of transformed human bronchial epithelial-cell strains Int J Oncol 1994 4 461 470 21566947 10.3892/ijo.4.2.461

[B24] Turkson J Bowman T Garcia R Caldenhoven E de Groot RP Jove R Stat3 activation by Src induces specific gene regulation and is required for cell transformation Mol Cell Biol 1998 18 2545 2552 9566874 10.1128/mcb.18.5.2545 PMC110634

[B25] Littlefield SL Baird MC Anagnostopoulou A Raptis L Synthesis, characterization and Stat3 inhibitory properties of the prototypical platinum(IV) anticancer drug, [PtCl3(NO2)(NH3)2] (CPA-7) Inorg Chem 2008 47 2798 2804 10.1021/ic702057q 18269242

[B26] Bromberg JF Wrzeszczynska MH Devgan G Zhao Y Pestell RG Albanese C Stat3 as an oncogene Cell 1999 98 295 303 10.1016/S0092-8674(00)81959-5 10458605

[B27] Anagnostopoulou A Vultur A Arulanandam R Cao J Turkson J Jove R Differential effects of Stat3 inhibition in sparse vs confluent normal and breast cancer cells Cancer Lett 2006 242 120 132 10.1016/j.canlet.2005.10.047 16377083

[B28] Andersson H Brittebo E Proangiogenic effects of environmentally relevant levels of bisphenol A in human primary endothelial cells Arch Toxicol 2012 86 465 474 10.1007/s00204-011-0766-2 22045264

[B29] Brownell HL Whitfield JF Raptis L Elimination of intercellular junctional communication requires lower Rasleu61 levels than stimulation of anchorage-independent proliferation Cancer Detect Prev 1997 21 289 294 9232318

[B30] Cole SP Campling BG Dexter DF Holden JJ Roder JC Establishment of a human large cell lung tumor line (QU-DB) with metastatic properties in athymic mice Cancer 1986 58 917 923 10.1002/1097-0142(19860815)58:4<917::AID-CNCR2820580419>3.0.CO;2-0 3719556

[B31] Anger B Bockman R Andreeff M Erlandson R Jhanwar S Kameya T Characterization of two newly established human cell lines from patients with large-cell anaplastic lung carcinoma Cancer 1982 50 1518 1529 10.1002/1097-0142(19821015)50:8<1518::AID-CNCR2820500812>3.0.CO;2-R 7116286

[B32] Xie HQ Huang R Hu VW Intercellular communication through gap junctions is reduced in senescent cells Biophys J 1992 62 45 47 10.1016/S0006-3495(92)81773-3 1600097 PMC1260479

[B33] Aleshin A Finn RS SRC: a century of science brought to the clinic Neoplasia 2010 12 599 607 20689754 10.1593/neo.10328 PMC2915404

[B34] Brownell HL Whitfield JF Raptis L Cellular Ras partly mediates gap junction closure by the polyoma virus middle Tumor antigen Cancer Lett 1996 103 99 106 10.1016/0304-3835(96)04187-0 8616815

[B35] Shen Y Khusial PR Li X Ichikawa H Moreno AP Goldberg GS SRC utilizes Cas to block gap junctional communication mediated by connexin43 J Biol Chem 2007 282 18914 18921 10.1074/jbc.M608980200 17488714

[B36] McLemore ML Grewal S Liu F Archambault A Poursine-Laurent J Haug J STAT-3 activation is required for normal G-CSF-dependent proliferation and granulocytic differentiation Immunity 2001 14 193 204 10.1016/S1074-7613(01)00101-7 11239451

[B37] Geletu M Chaize C Arulanandam R Vultur A Kowolik C Anagnostopoulou A Stat3 activity is required for gap junctional permeability in normal epithelial cells and fibroblasts DNA Cell Biol 2009 28 319 327 10.1089/dna.2008.0833 19456249

[B38] Theiss C Mazur A Meller K Mannherz HG Changes in gap junction organization and decreased coupling during induced apoptosis in lens epithelial and NIH-3T3 cells Exp Cell Res 2007 313 38 52 10.1016/j.yexcr.2006.09.029 17123514

[B39] Anagnostopoulou A Vultur A Arulanandam R Cao J Turkson J Jove R Role of Stat3 in normal and SV40 transformed cells Research Trends - Trends in Cancer Research 2006 2 93 103

[B40] Ozog MA Bernier SM Bates DC Chatterjee B Lo CW Naus CC The complex of ciliary neurotrophic factor-ciliary neurotrophic factor receptor alpha up-regulates connexin43 and intercellular coupling in astrocytes via the Janus tyrosine kinase/signal transducer and activator of transcription pathway Mol Biol Cell 2004 15 4761 4774 10.1091/mbc.E04-03-0271 15342787 PMC524725

[B41] Rajasingh J Bord E Hamada H Lambers E Qin G Losordo DW STAT3-dependent mouse embryonic stem cell differentiation into cardiomyocytes: analysis of molecular signaling and therapeutic efficacy of cardiomyocyte precommitted mES transplantation in a mouse model of myocardial infarction Circ Res 2007 101 910 918 10.1161/CIRCRESAHA.107.156786 17823373

[B42] Anagnostopoulou A Cao J Vultur A Firth KL Raptis L Examination of gap junctional, intercellular communication by in situ electroporation on two co-planar indium-tin oxide electrodes Mol Oncol 2007 1 226 231 10.1016/j.molonc.2007.06.002 19383296 PMC5543888

[B43] Greer S Honeywell R Geletu M Arulanandam R Raptis L housekeeping gene products; levels may change with confluence of cultured cells J Immunol Methods 2010 355 76 79 10.1016/j.jim.2010.02.006 20171969

