# Supplementary material for: Stat3 is a positive regulator of gap junctional intercellular communication in cultured, human lung carcinoma cells
Source: BMC Cancer. 2012 Dec 18;12:605. doi: 10.1186/1471-2407-12-605 (PMC3575370; doi:10.1186/1471-2407-12-605)
Supplement: Additional file 1 — Additional data. [file 1471-2407-12-605-S1.pdf]

Additional data

<u>Table Add-I</u>
Effect of Src downregulation upon Stat3-ptyr705

Cell line	Treatment[α]	Src[β] (%)	Stat3[β] (%)
**A549**	**DMSO**	95±11	93±12
"	**Dasatinib**	10±2	8±4
**E10-*Src***	**DMSO**	98±12	98±15
"	**Dasatinib**	8±1	10±1
**SK-LuCi6-*Src***	**DMSO**	100±12	100±12
"	**Dasatinib**	3±1	5±3
**SK-Lu1**	**DMSO**	85±5	90±11
"	**Dasatinib**	15±4	13±3
**CALU-1**	**DMSO**	96±9	100±10
"	**Dasatinib**	11±2	13±3
**SW-900**	**DMSO**	100±13	100±12
"	**Dasatinib**	12±2	14±3
**CALU-6**	**DMSO**	95±11	93±10
"	**Dasatinib**	8±2	10±3

[α]Cells were treated with 1μM Dasatinib, or the DMSO carrier when 50% confluent, for 24 hrs (see Methods).

[β] Stat3-tyr705 or Src-ptyr418 levels were measured by Western blotting, as in Table I, legend.

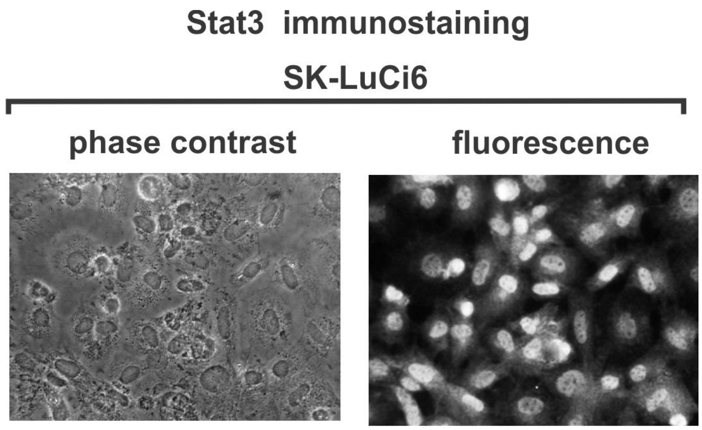

Figure Add-1:

SK-LuCi6 cells were fixed 4% paraformaldehyde, permeabilized in 0.2% Triton-X100 and probed with a Stat3 antibody (Cell Signalling, #9132 diluted at 1:100) followed by AlexaFluor-coupled, goat anti-rabbit IgG. Note that most of the staining is nuclear.
